# Effect of Fe^3+^ on the nutrient removal performance and microbial community in a biofilm system

**DOI:** 10.3389/fmicb.2023.1140404

**Published:** 2023-04-06

**Authors:** Tong Wu, Le Zhong, Ji-Wei Pang, Nan-Qi Ren, Jie Ding, Shan-Shan Yang

**Affiliations:** ^1^State Key Laboratory of Urban Water Resource and Environment, School of Environment, Harbin Institute of Technology, Harbin, China; ^2^China Energy Conservation and Environmental Protection Group, CECEP Talroad Technology Co., Ltd., Beijing, China

**Keywords:** Fe^3+^, nitrogen removal, microbial community assembly, species interactions, metabolic function prediction

## Abstract

In this study, the influence of Fe^3+^ on N removal, microbial assembly, and species interactions in a biofilm system was determined. The results showed that maximum efficiencies of ammonia nitrogen (NH_4_^+^-N), total nitrogen (TN), phosphorus (P), and chemical oxygen demand (COD) removal were achieved using 10 mg/L Fe^3+^, reaching values of 100, 78.85, 100, and 95.8%, respectively, whereas at concentrations of 15 and 30 mg/L Fe^3+^ suppressed the removal of NH_4_^+^-N, TN, and COD. In terms of absolute abundance, the expression of bacterial *amoA*, *narG*, *nirK*, and *napA* was maximal in the presence of 10 mg/L Fe^3+^ (9.18 × 10^5^, 8.58 × 10^8^, 1.09 × 10^8^, and 1.07 × 10^9^ copies/g dry weight, respectively). Irrespective of Fe^3+^ concentrations, the P removal efficiency remained at almost 100%. *Candidatus*_Competibacter (10.26–23.32%) was identified as the most abundant bacterial genus within the system. Determinism (50%) and stochasticity (50%) contributed equally to microbial community assembly. Co-occurrence network analysis revealed that in the presence of Fe^3+^, 60.94% of OTUs in the biofilm system exhibited positive interactions, whereas 39.06% exhibited negative interactions. Within the OTU-based co-occurrence network, fourteen species were identified as key microbes. The stability of the system was found to be predominantly shaped by microbial cooperation, complemented by competition for resources or niche incompatibility. The results of this study suggested that during chemical P removal in wastewater treatment plants using biofilm methods, the concentration of supplemental Fe^3+^ should be maintained at 10 mg/L, which would not only contribute to P elimination, but also enhance N and COD removal.

## 1. Introduction

Excessive phosphorus (P) in wastewater can cause serious pollution and drive eutrophication, causing severe danger to the environment and human health (Yang et al., 2022). Compared with physical and chemical technologies, biological technologies are commonly applied and relatively inexpensive, while their development in practical wastewater treatment plants (WWTPs) has been limited due to inconsistent P removal (Su et al., 2013; Cao et al., 2023). Therefore, to achieve efficient elimination of P, many WWTPs are supplemented with chemical P removal using metal salts (Liu B. et al., 2022).

Fe^3+^ is one of the metal ions used in chemical P removal (Ping et al., 2023). The presence of Fe^3+^ can promote a range of polymerization reactions leading to the formation of multinuclear hydroxyl complexes characterized by long linear structures, which can be removed *via* neutralization, adsorption bridging, and flocculation sweeping, followed by P removal *via* precipitation and separation (Ismail et al., 2022). In addition, as an indispensable constituent of numerous functional proteins (such as cytochromes and iron-sulfur and iron-nickel proteins), Fe is an essential element for microbial growth (Saoudi et al., 2022), and is active in cellular metabolic reactions (Wang et al., 2021). The findings of previous studies have indicated that Fe^3+^ enhances the particle size, absorption, and sedimentation properties of sludge (Zhang et al., 2021). Furthermore, Fe^3+^ has been demonstrated to contributes to the generation of certain anaerobic and anoxic zones in sludge, thereby enhancing the efficacy of simultaneous nitrification and denitrification (Ifelebuegu and Ojo, 2019; Campo et al., 2020). Wang et al. (2014) have examined the effect of Fe^3+^ on nitrogen (N) removal performance in a modified biological aerated filter, showing that nitrification performance was enhanced in the presence of Fe^3+^, whereas denitrification performance was inhibited. Furthermore, Jia et al. (2016) have demonstrated that the use of acetate as a carbon source in a laboratory-scale sequencing batch reactor contributed to promoting total nitrogen (TN) removal at an Fe^3+^ concentration of 40 mg/L. In contrast, in a batch reactor with citrate as the carbon source, 17.7 mg/L Fe ^3+^ was found to suppress the overall denitrification process (Ramírez et al., 2018). In addition, Pang and Wang (2023) have recently shown that at concentrations of 10 and 50 mg/L, Fe^3+^ can markedly suppress the removal of NO_3_^–^-N and facilitate the accumulation of NO_2_^–^-N during solid-phase denitrification. In their survey of the effects of Fe^3+^ on the performance of an A^2^O system, Zhang et al. (2021) observed a concentration-dependent effect, with chemical oxygen demand (COD) and ammonia nitrogen (NH_4_^+^-N) removal efficiencies being enhanced at Fe^3+^ concentrations lower than 10 mg/L, whereas these processes were effectively suppressed at Fe^3+^ concentrations higher than 10 mg/L. Collectively, these findings thus indicate that different concentrations of Fe^3+^ may exert differing effects in different treatment systems, thereby highlighting the necessity to comprehensively investigate the Fe^3+^-mediated removal of N. In this regard, it has been established that differences in the influence of Fe^3+^ on N removal in treatment systems are primarily attributable to changes in the structure and interrelationships of the associated microbial communities (Wang et al., 2021).

To elucidate the mechanisms underlying the effects of Fe^3+^ on N-removal performance, several researchers have characterized the diversity, structure, and functional characteristics of microbes in different systems (Wang et al., 2021; Zhang et al., 2021). Fe^3+^ has been established to influence the interrelationship among phosphate-accumulating organisms (PAOs) and those utilizing sugars, thereby influencing the removal of N and P from the system (Wang et al., 2015). Furthermore, Fe^3+^ has been found to modify the N cycle and promotes or inhibits the activity of certain microorganisms. Conversely, an increase in the abundance of certain nitrification-related enzymes or microbes has been observed in the presence of Fe^3+^, whereas this contributes to a reduction in denitrification efficiency (Huang et al., 2022). The assembly of microbial communities is associated with two important and complementary processes, namely deterministic processes based on the ecological niche theory, and stochastic processes based on the neutral theory (He et al., 2022; Lu M. et al., 2022). In deterministic processes, microbial community patterns are assumed to be controlled by environmental filtering and various biological interactions (Xiang et al., 2022; Zhang et al., 2022), whereas in stochastic processes, the roles of probabilistic diffusion and ecological drift are emphasized (Chen et al., 2022; Liu J. et al., 2022). In this context, the studies conducted to date have generally analyzed only the effects of Fe^3+^ on biological systems in terms of N and P removal efficiency and microbial community composition. Comparatively, few studies have evaluated microbial community assembly, functional genes, key microbes, and niche breadth within biofilm systems in the presence of Fe^3+^, which accordingly limits our understanding of N removal mechanisms.

The objectives of this study were to (i) elucidate the influence of Fe^3+^ on N removal efficiency in a biofilm system during simultaneous chemical P removal; (ii) identify the microbial community structure in biofilms, along with the metabolic pathways, functional genes, and expression of related enzymes; and (iii) determine the microbial community assembly process in biofilms and the interactions between species during chemical P removal. The findings of this study will provide theoretical support for the simultaneous removal of N and P using a biofilm system based on analyses of the variation in nutrients and microbes. Our findings will also contribute to enhancing the efficiency of WWTPs and thereby protection of the aquatic environment.

## 2. Materials and methods

### 2.1. Reactor setup

The experiments in this study were conducted using a reactor comprising a transparent plexiglass column with a valid volume of 5 L, containing a cross-flow honeycomb bionic microbial carrier. To simulate a septic tank discharge solution, we prepared a synthetic wastewater, which was pumped into the reactor from a 30 L holding tank using a peristaltic pump (BT100-2J, Longer). In addition, CH_3_COONa, NH_4_Cl, NaNO_2_, NaNO_3_, and KH_2_PO_4_ were added to the synthetic wastewater solution to provide sources of carbon, N, and P, whereas FeCl_3_⋅6H_2_O was added to provide influent Fe^3+^ concentrations of 10, 15, and 30 mg/L. The entire experiment was separated into five stages, depending on the influent Fe^3+^ concentration, as shown in Table 1. The pH was maintained at approximately 7.5 by adding NaHCO_3_. The influent was fed into the system using an adapted version of the sequencing batch method. The reaction was divided into three stages: anaerobic (240 min), aerobic (200 min), and anoxic (240 min). The system was operated two cycles per day, with each cycle lasting for 720 min, with a drainage ratio of 60%. The inoculated sludge was obtained from a well-operated reactor, forming a mixed liquor suspended solid concentration (MLSS) of approximately 3,500 mg/L after inoculation. After 36 h during which the sludge was in contact with the carrier, the suspended sludge in the reactor was discharged.

**TABLE 1:** Characteristics of the influent.

Parameters	Stage I (1–60d)	Stage II (61–100d)	Stage III (101–140d)	Stage IV (141–170d)	Stage V (171–210d)
Fe^3+^ (mg/L)	0	10	15	30	0
NH_4_^+^–N (mg/L)	75.43 ± 2.35	76.77 ± 3.65	74.98 ± 1.35	75.35 ± 3.09	75.67 ± 2.88
TN (mg/L)	78.83 ± 2.02	79.04 ± 1.65	78.96 ± 1.52	79.01 ± 3.54	78.72 ± 1.98
P (mg/L)	6.75 ± 0.73	6.68 ± 0.73	6.72 ± 0.95	6.75 ± 0.41	6.74 ± 0.86
COD (mg/L)	308.14 ± 2.43	311.32 ± 1.92	309.58 ± 4.32	310.47 ± 3.22	309.83 ± 2.83
pH	7.45–7.60	7.45–7.60	7.45–7.60	7.45–7.60	7.45–7.60

### 2.2. Analytical methods

Standard Methods for the Examination of Water and Wastewater were used to determine NH_4_^+^-N, COD, and P (American Public Health Association [APHA], 2005) concentrations. Dionex ICS-900 Ion Chromatography was used to analyze NO_2_^–^-N and NO_3_^–^-N. TN was considered the sum of NH_4_^+^-N, NO_2_^–^-N, and NO_3_^–^-N. MLSS was evaluated using standard procedures. DO and pH were monitored using DO and a pH meters (Rex, Shanghai), respectively.

### 2.3. Functional genes and microbial community

To elucidate microbial community structure and dynamics, five biofilm samples, differing with respect to Fe^3+^ concentration, were collected from the system on days 60 (D60), 100 (D100), 140 (D140), 170 (D170), and 210 (D210), respectively, for high-throughput sequencing and functional gene quantification. For comparative purposes, the initial sludge inoculum was defined as D0. Microbial DNA was extracted from samples using a GenElute™ 96 Well Tissue Genomic DNA Purification Kit (Merck, NJ, United States), and high-throughput sequencing of selected 16S rRNA genes for analysis of microbial communities was performed by Mayobio Biopharmaceutical Technology Ltd (Shanghai, China). The primers and specific amplification and sequencing steps were consistent with those described by Zhong et al. (2023). Functional genes were quantified based on fluorescence quantitative PCR using previously described primers (Wu et al., 2022) and the qPCR procedure described by Zhou et al. (2022).

### 2.4. Network construction

To investigate microbial interactions, a molecular ecological network analysis platform (iNAP)^1^ was used to establish microbial co-occurrence networks and perform partial statistical analysis calculations (Deng et al., 2012). The system network structure was constructed throughout the entire period of operation using the random matrix theory (RMT). Spearman’s correlation matrix analysis was performed using the operational taxonomic units (OTUs) in the top 50%. Random networks corresponding to each empirical network were generated after 100 calculations to verify the degree of randomness within the generated ecological network, and the topological characteristics of each random network were calculated. Gephi (0.9.5) was used to visualize the networks and identify microbial co-occurrence relationships. Four different ecological network node roles were determined: peripherals (Zi ≤ 2.5 and Pi ≤ 0.62), network hubs (Zi > 2.5 and Pi > 0.62), module hubs (Zi > 2.5 and Pi ≤ 0.62), and connectors (Zi ≤ 2.5 and Pi > 0.62) (Zhong et al., 2023). Depending on their respective topological roles, module hubs and connectors were considered key microbes.

## 3. Results

### 3.1. Effect of Fe^3+^ on nutrient removal

**FIGURE 1:**
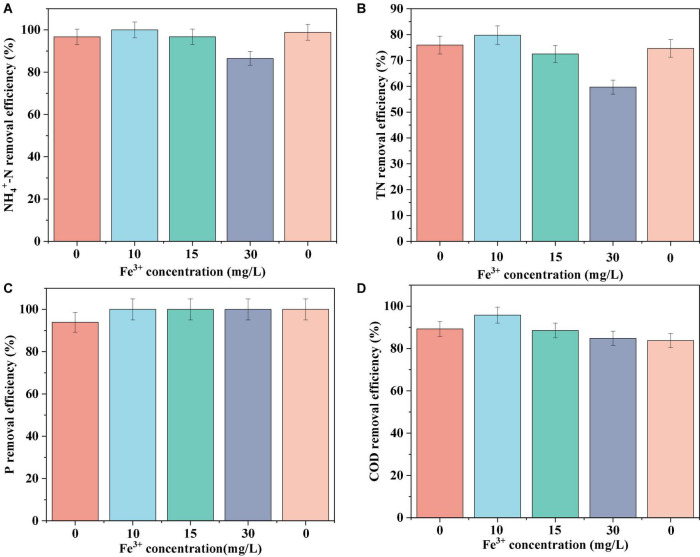
Average NH_4_^+^-N **(A)**, TN **(B)**, P **(C)**, and COD **(D)** removal efficiency of the system at different Fe^3+^ concentrations.

The N, P, and COD removal efficiencies of the biofilm system are shown in Figure 1. In the absence of Fe^3+^, the system achieved removal efficiencies of 96.72 ± 2.67, 75.05 ± 0.98, 93.92 ± 2.26, and 89.29 ± 1.46% for NH_4_^+^-N, TN, P, and COD, respectively. In response to the addition of 10 mg/L Fe^3+^ to the influent, the nutrient removal efficiency of the system reached a maximum level, achieving 100% NH_4_^+^-N removal, and increases of 3.8 and 6.51% in the removal of TN and COD, respectively. However, with a further increase in the concentration of Fe^3+^, we detected gradual reductions in the efficiencies with which NH_4_^+^-N, TN, and COD were removed. An influent Fe^3+^ concentration of 30 mg/L coincided with the lowest system performance, with average NH_4_^+^-N, TN, and COD removal efficiencies of 86.5, 59.69, and 84.83%, respectively. Accordingly, we established that whereas the addition of 10 mg/L Fe^3+^ can contribute to promoting the removal of N, P, and COD in a biofilm system, the addition of 15 or 30 mg/L Fe^3+^ had an inhibitory effect on removal. Following the cessation of Fe^3+^ dosing, we detected a recovery in N removal efficiency, whereas we observed no appreciable changes in COD removal efficiency. Notably, in response to Fe^3+^ supplementation, the efficiency with which P was removed increased to 100% and remained essentially stable at this level.

### 3.2. Community diversity

The data presented in Table 2 show that the OTU coverage index values of the seed sludge (D0) and all five system samples were higher than 0.98, thereby verifying that the sequencing results were sufficiently representative of the entire biofilm microbial community (Sang et al., 2020). To assess the diversity and richness of the microbial community, we used the Shannon diversity and ACE estimator indices, respectively (Xu et al., 2021; Zeng et al., 2021). Shannon index values were found to be the highest at D0, after which values underwent a continual decline until no further significant changes were observed from D60 to D210. In contrast, values obtained for the ACE index gradually increased from 1,573.36 at D0 to 1,688.67 at D100, although thereafter, they declined from 1,605.776 (D140) to 1,493.25 (D170), followed by a slight increase to 1,560.33 following the cessation of Fe^3+^ dosing (D210). These findings thus indicate that 10 mg/L Fe^3+^ contributed to enhancing community richness, whereas higher concentrations of Fe^3+^ (15 and 30 mg/L) were established to have suppressive effects.

**TABLE 2:** α-diversity of different samples.

Samples	Coverage	Shannon	ACE
D0	0.989	5.848	1573.361
D60	0.987	5.525	1627.889
D100	0.986	5.578	1688.674
D140	0.987	5.588	1605.776
D170	0.989	5.497	1493.252
D210	0.988	5.394	1560.332

**FIGURE 2:**
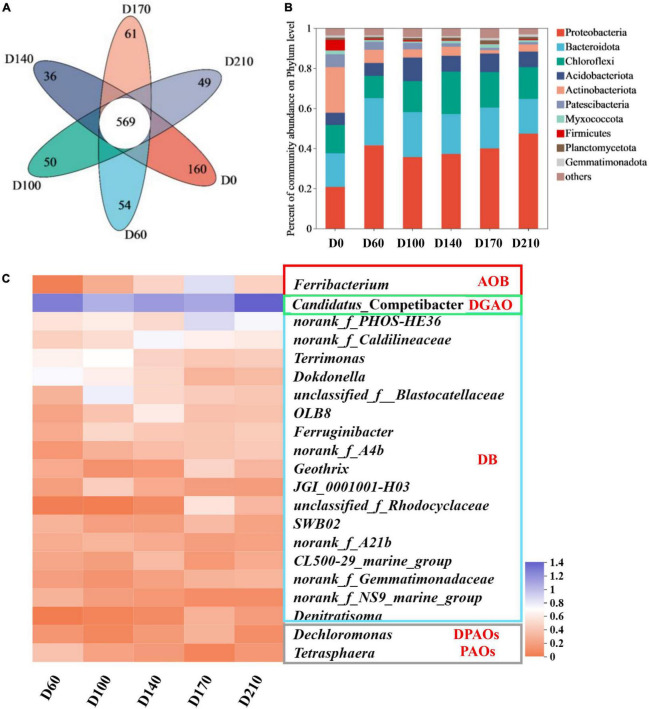
Venn diagram **(A)** and changes in species abundance at the phylum level **(B)**; variation in bacteria with nitrogen and phosphorus removal functions among the top 50 genera **(C)**.

As illustrated in Figure 2A, 569 OTUs were detected among the six samples. The seed sludge (D0) was found to contain 160 specific species and we detected considerably lower numbers of these species in the other five samples, ranging from 36 to 61 OTUs. These smaller numbers of specific species in the samples provided evidence to indicate that by influencing a small number of key microbes, the presence of Fe^3+^ affected the entire microbial community.

### 3.3. Dynamics of N- and P-related microbes

Figure 2B shows that Proteobacteria, a phylum containing many functional N- and P-removing genera (Wu et al., 2022), was the most abundant in all biofilm samples (35.68–47.36%). The second most abundant phylum was Bacteroidota (17.44–23.47%), an common marine bacteria, which play an essential role in the degradation of organics, followed by Chloroflexi (11.21–21.26%). Comparatively, the top three phyla in D0 were Acidobacteriota (22.73%), Proteobacteria (20.84%), and Bacteroidota (16.70%), indicating that as a consequence of domestication, there was a transition in microbial composition in the reactor, which is consistent with our findings with respect to community diversity.

Among the top 50 genera (Figure 2C), 21 were established to be associated with N and P removal, including one ammonia-oxidizing bacteria (AOB), one denitrifying glycogen-accumulating organism (DGAO), one PAO, one denitrifying PAO (DPAO), and seventeen denitrifying bacteria (DB). In all five biofilm samples, *Candidatus*_Competibacter was the most abundant genus, with relative abundances of 17.37% (D60), 10.26% (D100), 12.89% (D140), 11.32% (D170), and 23.32% (D210), respectively. Bacteria within the genus *Candidatus*_Competibacter are DGAOs that can convert carbon sources to polyhydroxyalkanoates [PHAs: poly-β-hydroxybutyrate (PHB) and poly-β-hydroxyvalerate (PHV)] and glycogen (Gly) for intracellular storage under anaerobic conditions and releasing them for endogenous denitrification under anoxic conditions (Hu et al., 2021). In response to the addition of 10 mg/L Fe^3+^, we detected a slight decline (0.91%) in the abundance of *Ferribacterium*, a genus containing nitrifying bacteria (Guo et al., 2022) (relative abundance of 1.05% at D60), whereas higher abundances were observed with increasing Fe^3+^ concentrations (maximum abundance of 6.09% at D170) and a rapid reduction in abundance (1.92%) was detected after Fe^3+^ had been withdrawn from the system. These findings would thus tend to indicate that Fe^3+^ may promote the nitrification capacity of the system. Among the 21 genera characterized by denitrification activity, *norank_f_PHOS-HE36*, *norank_f_Caldilineaceae*, *Terrimona*, *Dokdonella*, and *unclassified_f_Blastocatellaceae* (Sheng et al., 2021; Yao et al., 2022) were identified as being the most abundant (>1%). In the presence of 30 mg/L Fe^3+^, the relative abundance of *norank_f_PHOS-HE36* peaked at 6.36%, which was approximately 2. 38-, 2. 03-, and 2.83-fold higher than that recorded at D60, D100, and D140, respectively, and subsequently declined to 4.57% following the cessation of Fe^3+^ dosing. *norank_f_Caldilineacea* reached a maximum relative abundance of 4.58% in response to the addition of 15 mg/L Fe^3+^, with abundances gradually declining concomitant with an increase Fe^3+^ concentration. *Terrimonas*, *Dokdonella*, and *unclassified_f_ Blastocatellaceae* were characterized by maximum abundances of 3.95, 3.19, and 5.01%, respectively, in the presence of 10 mg/L Fe^3+^, with abundances in each case declining thereafter with increasing Fe^3+^ concentrations. However, the abundances of *Terrimonas* and *Dokdonella* increased by 0.12 and 0.21%, respectively, after the removal of Fe^3+^ from the influent, whereas that of *unclassified_f_Blastocatellaceae* continued to decline to 1.57%, thereby indicating that different denitrifying bacteria have differing Fe^3+^ requirements. In terms of the total abundances of denitrifying microorganisms in each of the five assessed system stages, we obtained values of 21.91% (D60), 29.30% (D100), 25.34% (D140), 28.61% (D170), and 24.47% (D210), thus indicating that supplementation of synthetic wastewater with 10 mg/L Fe^3+^ can contribute to enhancing the denitrification performance of biofilm systems. Among other genera, we found that *Dechloromonas*, a DPAO that removes P *via* denitrification during the anoxic phase, using NO_2_^–^-N or NO_3_^–^-N as electron donors (Zhao et al., 2022), exhibited a maximum relative abundance (1.04%) at 30 mg/L Fe^3+^, whereas in response to the addition of Fe^3+^, we detected a reduction in the abundance of *Tetrasphaera* (0.17–1.45%), a PAO that uses oxygen as an electron donor to remove P under aerobic conditions (Li et al., 2022).

**FIGURE 3:**
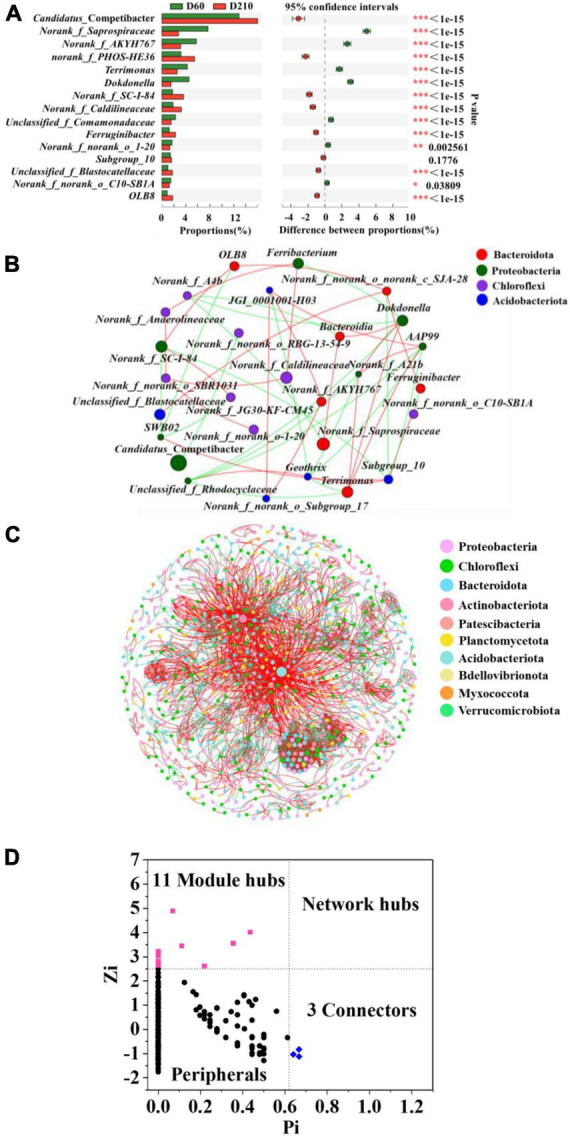
Fisher’s test at the genus level for samples from D60 and D210 **(A)**, co-occurrence network based on phylum level **(B)**, co-occurrence network based on operational taxonomic units (out) level **(C)**, and Zi-Pi plot **(D)**.

To determine the specific differences among microbial communities within the biofilm system in response to the addition of Fe^3+^ and following its cessation, we performed Fisher exact test analyses at the genus level using samples D60 and D210 (Figure 3A). A total of 14 genera were found differ significantly between the two samples, including certain denitrifying bacteria, such as *norank_f_PHOS-HE36*, *Terrimonas*, *Dokdonella*, *OLB8*, and *Ferruginibacter*. These findings accordingly indicate that the biofilm microbial community did not completely recover to its former levels following the cessation of Fe^3+^ supplementation.

### 3.4. Microbial interactions and key microbes

Throughout the experiment, Fe^3+^ in the influent was considered an environmental factor, as the concentration of Fe^3+^ in actual wastewater often undergoes considerable fluctuation over time. Consequently, to further enhance the practical applicability of our findings, we combined steady-state microbial samples from different stages of the treatment process with the aim of constructing co-occurrence networks, and thereby elucidate the interactions among microbes, and thus identify key microbes. Microbial interactions were categorized as either positive (mutualistic and commensalistic) or negative (competitive, predatory, and amensalism) (Deng et al., 2016). As shown in Figure 3B, Table 3, we also detected difference among the microbes interacted with the different genera. For example, the most abundant genus, *Candidatus*_ Competibacter was characterized by only a single negative correlation with *norank_f_norank_o_RBG-13-54-9*, whereas the second most abundant genus, *norank_f_Saprospiraceae*, was positively correlated with *Bacteroidia* and negatively correlated with *norank_f_A4b*. Furthermore, the denitrifying bacterium *Terrimonas* was found to be directly correlated with five genera, with which there three positive interactions (*norank_f_A21b*, *AAP99*, and *Dokdonella*) and two negative interaction (*Geothrix* and *unclassified_f_Rhodocyclaceae*). In contrast, the denitrifier *OLB8* was positively correlated with *norank_f_SC-I-84* and *norank_f_norank_o_c_SJA-28*, and negatively correlated with *SWB02*.

**TABLE 3:** Topological properties of OTU-based co-occurrence networks.

	Empirical network indexes	100 Random network indexes
Total nodes	961	–
Total links	3,512	–
R^2^ of power-law	0.921	–
Average degree (avg K)	7.309	–
Average clustering coefficient (avg CC)	0.558	0.017 ± 0.003
Harmonic geodesic distance (HD)	3.337	3.018 ± 0.014
Transitivity (trans)	0.382	0.056 ± 0.003
Modularity (Fast_greedy)	0.728	0.319 ± 0.004
σ = (CC/CCr)/(HD/HDr) = 29.686		

Microbial interactions are essential for maintaining system stability, and to identify microbial interactions within the biofilm system community, further analysis was performed on the interactions among OTUs. As shown in Figure 3C, 1,130 nodes were initially used to construct the network, which eventually contained 961 nodes (85% of the initial total number of nodes), providing an indication of the size and complexity of the network. A total of 3,512 edges were identified in the network, with positive and negative correlations of approximately 60.94 and 39.06%, respectively. In addition, we identified a small world in the network, as indicated by a σ value of 29.686 (Xu et al., 2021).

A total of 14 key microbes were identified in the network based on previous literature reports (Figure 3D). The relative abundances and respective genera and phyla of which are presented in Table 4. Among these key microbes, nine were present at low abundances, whereas the remaining five OTUs were present at relatively high abundances. The key microbes with high abundance OTUs were OTU1123, OTU1230, OTU2161, OTU1200, and OTU2435. Among these, OTU1123 is a bacterium in the genus *Candidatus*_Competibacter, a group of DGAOs that can release intracellular carbon sources for denitrification in the absence of electron acceptors. OTU2161 belongs to *norank_f_A4b* bacterium in class Anaerolineae, the members of which play roles in polysaccharide degradation and carbohydrate fermentation (Zhang et al., 2023). OTU1200 and OTU2434 are, respectively members of the taxa *norank_f_norank_ o_ norank_c_OLB14* and *Geothrix*, both of which play denitrifying roles (Ma et al., 2021). Among the species with lower relative abundance, OTU689 (0.03126%) is a species of *SM1A02*, a genus with good nitrification capacities and anammox potential (Wan et al., 2022). As key microbes, these species are assumed to perform essential functions in maintaining the overall functional stability of the system.

**TABLE 4:** Key microbes identified in operational taxonomic units (out)-based co-occurrence networks.

Module hubs	Abundance (%)	Genus	Phylum
OTU280	0.00485	*MND1*	Proteobacteria
OTU689	0.03126	*SM1A02*	Planctomycetota
OTU1103	0.02533	*norank_f_norank_o_Saccharimonadales*	Patescibacteria
OTU1123	0.99048	*Candidatus*_Competibacter	Proteobacteria
OTU1230	0.15520	*norank_f_norank_o_norank_c_Dojkabacteria*	Patescibacteria
OTU1355	0.00377	*norank_f_Polyangiaceae*	Myxococcota
OTU1455	0.00377	*unclassified_k_norank_d_Bacteria*	unclassified_k_norank_d_Bacteria
OTU1680	0.01940	*norank_f_Microscillaceae*	Bacteroidota
OTU1703	0.00485	*norank_f_norank_o_Candidatus*_ Peregrinibacteria	Patescibacteria
OTU2161	0.12017	*norank_f_A4b*	Chloroflexi
OTU2386	0.00539	*Anaeromyxobacter*	Myxococcota
**Connectors**			
OTU565	0.00593	*norank_f_Bacteroidetes_vadinHA17*	Bacteroidota
OTU1200	0.19077	*norank_f_norank_o_norank_c_OLB14*	Chloroflexi
OTU2435	0.18053	*Geothrix*	Acidobacteriota

### 3.5. Microbial community assembly and niche breadth

**FIGURE 4:**
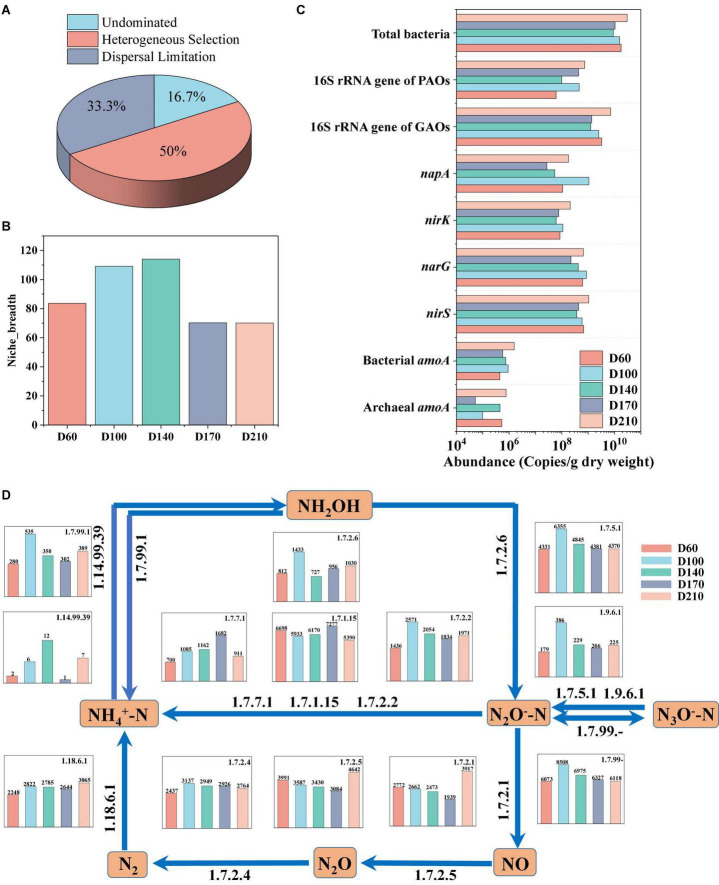
Bacterial assembly of biofilm community **(A)**, variation in niche breadth over time **(B)**, changes in the absolute abundance of functional genes **(C)**, and the nitrogen metabolism and relative abundance of coding genes for key enzymes based on PICRUSt prediction **(D)**.

The assembly of the microbial community in biofilms was investigated using the null model. The mechanisms underlying community development are considered to be either deterministic or stochastic processes (Ya et al., 2022). Deterministic processes include homogeneous and heterogeneous selection, indicating that the entire system is influenced by interspecific interactions or other circumstantial factors (Hu et al., 2020). Stochastic processes consider all microorganisms to be ecologically equivalent, involving random birth, death, dispersal, extinction, and species formation, and can mainly be categorized as dispersal limitation, homogenizing dispersal, or undominated (Zhou and Ning, 2017). In the present study, we identified three predominant processes, among which, heterogeneous selection accounted for 50% of the overall microbial community assembly processes, dispersal limitation accounted for 33.3% of the assembly processes, and the remaining influence was undominated (Figure 4A). These findings accordingly indicate that the assembly of microbial communities is mediated *via* both deterministic and stochastic processes. For each operational stage in the treatment process, we also characterized the microbial community in terms of Levins niche breadth index (Zhang et al., 2018), obtaining an index value of 83 at D60, which increased to values higher than 100 at influent Fe^3+^ concentrations of 10 and 15 mg/L (Figure 4B). However, in response to the addition of 30 mg/L Fe^3+^, the niche breadth index value dropped to 70 and showed no significant change after the cessation of Fe^3+^ dosing.

### 3.6. Functional predictions

**FIGURE 5:**
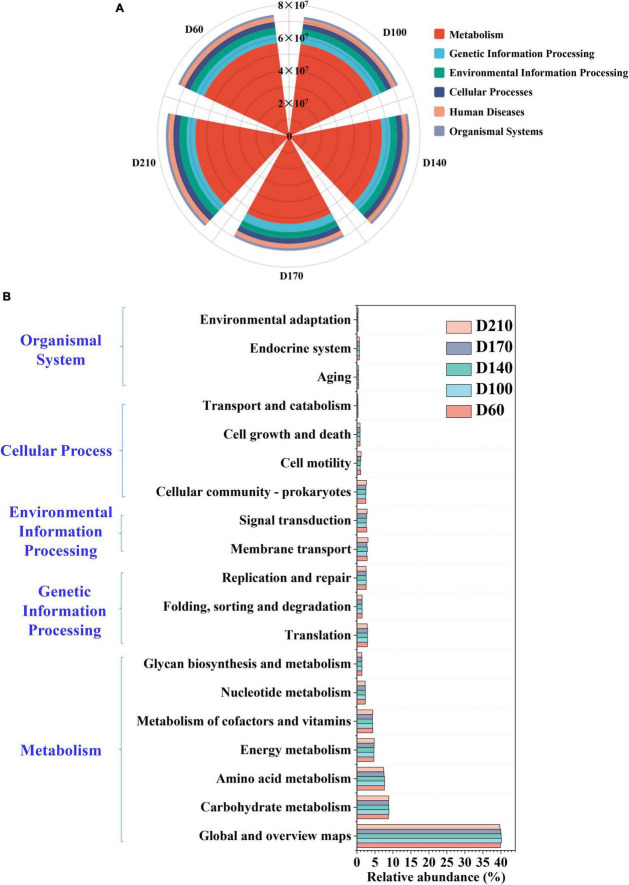
The functional prediction of microbial community based on the Kyoto Encyclopedia of Genes and Genomese (KEGG) database on Level 1 **(A)** and Level 2 **(B)**.

To analyze microbial community dynamics in greater depths, the PICRUSt was used to make functional predictions for all five samples. The predicted results were compared with the Kyoto Encyclopedia of Genes and Genomese (KEGG) database to interpret the predicted changes in microbial function and the enzymes associated with related to N metabolism. Mechanistic processes were found to be the dominant category, accounting for more than 75% of each sample, although the proportion declined as the experiment proceeded (Figure 5A). Figure 5B shows the abundances of 19 core metabolic pathways based on Level 2. Global and overview maps were the primary metabolic pathways, reaching a relative abundance of 39.90% at D60 and a maximum abundance of 40.16% with the addition of 10 mg/L Fe^3+^, followed by a gradual decrease thereafter. The relative abundance of carbohydrate metabolism was also the highest in D100 (8.90%), showing an increase of approximately 0.16% compared to D60. Amino acid metabolism was ranked third overall, with relative abundances of 7.66, 7.71, 7.67, 7.52, and 7.40% at D60, D100, D140, D170, and D210, respectively, which were similar to the trends observed in the global and overview maps. Similarly, nucleotide metabolism, glycan biosynthesis, metabolism, and replication and repair also exhibited maximal abundance in the presence of 10 mg/L Fe^3+^, reaching 2.37, 1.43, and 2.59, respectively.

For proteins to undergo oxidation oxidized to CO_2_ and H_2_O, they must initially be hydrolyzed. Thus, the changes in the relevant enzymes during N metabolism were investigated, as shown in Figure 4D. Thirteen N-conversion-related enzymes have been identified. During N conversion, hydroxylamine is converted into NH_4_^+^-N (EC1.7.99.1), NO_2_^–^-N (EC1.7.2.6), and NO_3_^–^-N (EC1.7.99-) by hydroxylamine oxidation. Compared with D60, the oxidative capacity of hydroxylamine was the strongest in the presence of 10 mg/L Fe^3+^. The stimulation caused by the addition of 10 and 15 mg/L Fe^3+^ contributed to a reduction in hydroxylamine, whereas the addition of 30 mg/L Fe^3+^ had an inhibitory effect on this reduction (EC1.14.99.39). NO_3_^–^-N was converted to NO_2_^–^-N *via* EC1.9.6.1 and EC1.7.5.1, and the abundance of both enzymes was significantly enhanced in the presence of 10 mg/L Fe^3+^. The addition of Fe^3+^ inhibited both EC1.7.2.1 and EC1.7.2.5, thereby contributing to a reduction in the conversion of NO_2_^–^-N to NO and N_2_O (Huang et al., 2022). In contrast, both the conversion of N_2_O to N_2_ and N fixation were enhanced in response to the addition of Fe^3+^, whereas N_2_O was transformed to NH_4_^+^-N in the presence of EC1.7.7.1, EC1.7.1.15, and EC1.7.2.2. Furthermore, the abundances of EC1.7.7.1 and EC1.7.2.2 increased in all Fe^3+^ concentrations, whereas the abundance of EC1.7.1.15 increased in response to the presence of 30 mg/L Fe^3+^, but was reduced at Fe^3+^ concentrations of 10 and 15 mg/L. In addition, EC1.7.7.1 promoted ferredoxin-nitrite reductase, the abundance of which was proportional to the concentration of Fe^3+^.

### 3.7. Variation in functional genes associated with N and P removal

N and P are common nutrients in wastewater, the high levels of which can contribute to the eutrophication of aquatic environments, thereby posing a significant risk to ecological health (Fan et al., 2022). Therefore, the effect of Fe^3+^ on genes associated with N and P removal was analyzed (Figure 4C). It was accordingly found that the absolute abundance of functional genes within the total bacterial community declined in the presence of Fe^3+^ (from 9.02 × 10^9^ to 1.55 × 10^10^ copies/g dry weight) although it increased to 3.03 × 10^10^ copies/g dry weight after the cessation of Fe^3+^ supplementation. The absolute abundance of the 16S rRNA gene of GAOs was the highest at D60, reaching 3.26 × 10^9^ copies/g dry weight, declining with the addition of Fe^3+^, and recovering to 7.16 × 10^9^ copies/g dry weight after the cessation of Fe^3+^ dosing. Levels of the 16S rRNA gene of PAOs were the lowest at D60, reaching only 6.08 × 10^7^ copies/g dry weight, but increased to 4.56 × 10^8^ copies/g dry weight with the addition of 10 mg/L Fe^3+^, after which there was a gradual decline at higher Fe^3+^ concentrations prior to a further increase to 7.33 × 10^8^ copies/g dry weight with the cessation of Fe^3+^ dosing. In terms of the genes associated with N removal, compared with D60, D140, and D210, bacterial *amoA*, *narG*, *nirK*, and *napA* genes were all found to have the highest expression levels in the presence of 10 mg/L Fe^3+^ (9.18 × 10^5^, 8.58 × 10^8^, 1.09 × 10^8^, and 1.07 × 10^9^ copies/g dry weight, respectively). The expression of all four genes was higher in the D210 samples than in D60 samples, indicating that Fe^3+^ supplementation had the effect of promoting increases in the absolute abundances of these relevant functional genes.

## 4. Discussion

### 4.1. Effect of Fe^3+^ on nutrient removal

In this study, the highest N and COD removal efficiencies were observed with 10 mg/L Fe^3+^. A possible reason for this is that Fe^3+^ can stimulate the activity of enzymes related to N removal and can alter the composition of cells, acting as an active site for cytochromes, as well as being an important nutrient for microbial growth and metabolism (Chen and Strous, 2013; Wang et al., 2021). Zhang et al. (2021) studied the effects of different Fe^3+^ concentrations on the A^2^O process and accordingly detected the highest N and P removals in the presence of 10 mg/L Fe^3+^, which is consistent with our findings in the present study. However, at Fe^3+^ concentrations in excess of 10 mg/L, we detected reductions in the N and COD removal capacities of the system, which is assumed to reflect the fact that elevated levels of Fe^3+^ can promote the oxidation of oxygen within microbial cells, thereby generating oxygen or hydroxyl radicals and thus causing cellular damage and inactivation (Mena et al., 2011). In the presence of Fe^3+^, we found the P removal efficiency of the system to be maintained at a stable level of approximately 100%, which could be ascribed to the fact that the Fe^3+^ provided in this study was applied in the form of FeCl_3_⋅6H_2_O, as thus the Fe^3+^ on biofilms would be present as [Fe(H_2_O)_6_]^3+^. A single Fe^3+^ ion can produce an average of six adsorption vacancies, thereby facilitating bonding with other substances, and consequently resulting in flocculation in response to the effect of adsorption bridging (Zhang et al., 2021). However, Fe^3+^ can combine with PO_4_^3–^ and HPO_4_^2–^ to form precipitates, thereby facilitating the chemical removal of P, and thus the effective elimination of P from wastewater (Huang et al., 2022).

### 4.2. Response of microbes to Fe^3+^

Microorganisms are the main drivers of biofilm processes, for which Fe^3+^ is an essential nutrient (Yuan et al., 2021). In the present study, we examined the effects of Fe^3+^ on the structure and function of microbes and key functional genes. The interactions among microorganisms and community assembly processes in the presence of Fe^3+^ were analyzed using molecular ecological networks and the null model. Although to a certain extent the concentration of Fe^3+^ was found to influence microbial abundance at the phylum level, the dominance of the three most abundant phyla appeared to be concentration independent. Among the 50 most abundant genera, the only bacteria with known nitrification function are those within the genus *Ferribacterium*, the predominance of which was reduced in the presence of 10 mg/L Fe^3+^, whereas no ammonia-oxidizing bacteria were detected, which tends to be inconsistent with the observed high ammonia removal performance. We suspect that this anomaly could be attributed to the activity of certain bacteria present at low abundance that have ammonia N oxidation functions, or the co-occurrence of archaea with ammonia N oxidation capacity. The qPCR results indicated that the absolute abundance of bacterial *amoA* (9.18 × 10^5^ copies/g dry weight) was the highest in the presence of 10 mg/L Fe^3+^, and the absolute content of archaeal *amoA* was only 9.80 × 10^4^ copies/g dry weight. However, the sum of the absolute abundances of archaeal and bacterial *amoA* was established to be the highest in all five phases of the treatment process. These findings thus tend to indicate the presence of certain other microbes with ammonia oxidation functions, thereby accounting for the higher NH_4_^+^-N removal efficiency at this stage (stage IV). In addition to D210, the relative abundance of *Dechloromonas* and *Tetrasphaera* and the absolute abundance of the 16S rRNA gene of PAOs also peaked at 10 mg/L and thereafter declined, although the P removal efficiency remained at 100%, thereby further indicating that a certain proportion of P in the system was removed *via* chemisorption precipitation. Significant changes were observed in the abundance of functional genera associated with N and P removal at different Fe^3+^ concentrations, which thus provides evidence to indicate differences in microbial requirements for Fe^3+^. Moreover, we found that even when Fe^3+^ supplementation was terminated, microbial activity did not completely return to the initial levels in the absence of Fe^3+^, and indeed, we detected an increase in the abundance of genes associated with N and P removal at D210. These findings are consistent with the N removal efficiency results, thus indicating that the effect of Fe^3+^ on the system is reversible.

Species with a wide niche breadth are able to survive in a diverse range of environments and are assumed to be more competitive, whereas a narrower niche breadth is taken to be indicative of a species that is at a competitive disadvantage (Wilson and Hayek, 2015). As indicated by the change in niche breadth in the presence of 10 mg/L Fe^3+^, we detected a wider niche breadth than that at D60, thereby signifying pronounced interspecies competitiveness at this stage. Furthermore, although Shannon index values remained almost unchanged, those of the ACE index increased in response to the addition of 10 mg/L Fe^3+^, thereby providing further evidence to indicate that the increase in niche breadth was associated with an increase in the abundance of more competitive species promoted by Fe^3+^. A continuation of the increase in niche breadth was detected in the presence of 15 mg/L Fe^3+^, and whereas Shannon index values remained largely unchanged, we observed reductions in those of the ACE index, thereby implying that competitiveness at this point may be a consequence of niche incompatibility. However, a contraction in niche breadth was detected in response to the addition of 30 mg/L and after Fe^3+^ was withdrawn, thereby indicating that high concentrations of Fe^3+^ have the effect of reducing interspecies competitiveness. We suspect that this trend can be ascribed to the inhibitory effects of high concentrations of Fe^3+^ on certain competitive species, and a transition to preferential cooperation, which remained the predominant theme following the withdrawal of Fe^3+^.

Co-occurrence network analysis at the OTU level revealed that 60.94% of relationships in the biofilm system were positively correlated during the removal of N and P from wastewater containing fluctuating concentrations of Fe^3+^. This predominance of positive correlations indicates that the relationships among microorganisms tend to be cooperative (Zhong et al., 2023). In this regard, the findings of previous studies have revealed that cooperative associations among microorganisms can be classified as either indirect facilitation, *via* cross-feeding or co-metabolism, or direct interactions between cells during co-aggregation or co-colonization (Faust and Raes, 2012). In contrast, 39.06% of the relationships were found to be negatively correlated, indicating the occurrence of predation, competition, and amensalism among microbes (Faust and Raes, 2012). Given that OTU-based co-occurrence networks only consider the interrelationships between bacteria, without taking into account ciliates or phages (Pernthaler, 2005), and high nutrient concentrations were present in the synthetic wastewater, we suspect that the negative correlations detected in the present study could be attributable to direct competition for resources or indirectly to incompatible niches, rather than from direct predation (Deng et al., 2016). Overall, however, the co-occurrence network analysis revealed that under conditions of fluctuating influent Fe^3+^ concentrations, the relationships among microorganisms were dominated by cooperation and supplemented by competition for resources or niche incompatibility, with these interactions complementarily maintaining the stability of the system. Moreover, we identified 14 microorganisms that appeared to play essential roles in maintaining the stability of the network, some of which were present at low abundance. This is consistent with the previous findings reported by Lu Z. et al. (2022), who found that microorganisms present at a low abundance may play roles that are comparable to or even of greater importance than those played by species characterized by a higher abundance.

### 4.2. The potential application

Typically, when treating wastewater with a low C/N ratio, an additional carbon source is often added to ensure adequate denitrification (Li et al., 2023). However, carbon source supplementation necessitates the use of large amounts of chemicals, the production of which generally involves the consumption of energy derived from fossil fuels, which to some extent could be construed as the transference of water pollution to air pollution, and consequently, does not contribute extensively to solving fundamental environmental problems (He et al., 2019). The findings in this study indicate that the supplementation of biofilm systems with Fe^3+^ to can effectively enhance the removal of N, P, and COD from municipal wastewater. Moreover, as the second most abundant metal ion in the Earth’s crust, Fe is a readily available and abundant resource. Consequently, Fe^3+^ can be added artificially to enhance the efficacy of low C/N ratio wastewater treatment, or alternatively, municipal wastewater can be mixed with industrial wastewater containing Fe^3+^ at a certain ratio to introduce Fe^3+^, thereby obtaining sufficient effluent quality. This not only reduces the cost of a supplemental carbon source and economic expenditure but also contributes to reducing energy consumption during the wastewater treatment process, which is of considerable importance from the perspective of protecting the aquatic environment. In the future, with a perspective toward optimizing the treatment process, the influence of environmental factors such as temperature, pH, feed water loading, and operation should be studied in greater depth.

## 5. Conclusion

In this study, a biofilm system was used to assess the effects of Fe^3+^ on wastewater nutrient removal efficiency and microbial community assembly. The results revealed that maximum NH_4_^+^-N, TN, P, and COD removal efficiencies were achieved in the presence of 10 mg/L Fe^3+^, reaching 100, 78.85, 100, and 95.8%, respectively. Fe^3+^ contributed to P removal by facilitating chemical precipitation, with the P removal efficiency of the system being consistently maintained at a level of almost 100% in the presence of different Fe^3+^ concentrations. Highest values for the absolute abundance of bacterial *amoA*, *narG*, *nirK* and *napA* (9.18 × 10^5^, 8.58 × 10^8^, 1.09 × 10^8^, and 1.07 × 10^9^ copies/g dry weight, respectively) were detected in response to supplementation with 10 mg/L Fe^3+^. Among the identified taxa in the biofilm microbial community, *Candidatus*_ Competibacter (10.26–23.32%) was found to be the most abundant genus within the system, and *norank_f_PHOS-HE36* was found to show good adaptability to high concentrations of Fe^3+^. The null model further confirmed the equal contribution of both determinism (50%) and stochasticity (50%) functions in the assembly of microbial communities. Co-occurrence network analysis revealed that 60.94% of the detected OTUs were engaged in positive interactions, whereas negative interactions accounted for only 39.06% of these OTUs. Fourteen OTUs were established to play key roles in the microecological environment of the reactor. The relationships among microbes were found to be predominantly cooperative, although we also found evidence to indicate competition for resources or niche incompatibility, which contribute to maintaining the overall stability of the biofilm system. Overall, the findings of this study indicate that for effective chemical P removal using biofilm systems, the concentration of supplemental Fe^3+^ should be maintained at 10 mg/L, which not only contributes to P removal but also enhances the elimination of N and COD.

## Data availability statement

The data presented in this study are deposited in the NCBI repository, accession number PRJNA933385.

## Author contributions

TW: data curation, writing, software, conducting the experiment and development of methods, and validation. LZ: resources, conceptualization, data collection, and writing. J-WP: conceptualization and methodology. N-QR: methodology and data collection. JD: writing, supervision, methodology, and funding acquisition. S-SY: data collection, supervision, funding, methodology, investigation, and reviewing. All authors contributed to the article and approved the submitted version.

## Footnotes

1
http://mem.rcees.ac.cn
